# Tinnitus burden in patients with chronic suppurative otitis media undergoing surgery in Nepal: a cross-sectional study

**DOI:** 10.1017/S0022215126105209

**Published:** 2026-08

**Authors:** Ruth Capper, Ram Bohara, Govind Bohara, Emma Stapleton

**Affiliations:** 1 Department of Otolaryngology, Doncaster Royal Infirmary, UK; 2 The Britain Nepal Otology Service, Nepal; 3 B.N. Hospital, Ganeshman Chowk, Nepalgunj, Nepal; 4 2. B.N. Hospital, Ganeshman Chowk, Nepalgunj, Nepal; 5 Manchester Academic Health Sciences Centre, The University of Manchester Faculty of Biology Medicine and Health, UK; 6 Department of Otolaryngology, Manchester Royal Infirmary, UK

**Keywords:** hearing loss, Nepal, quality of life, tinnitus

## Abstract

**Objectives:**

Tinnitus is a common and potentially distressing symptom associated with chronic ear disease. Its burden in patients with chronic suppurative otitis media in low-resource settings is not well characterised.

**Methods:**

A cross-sectional study was conducted during an ear surgery camp in Nepal. One hundred patients with chronic suppurative otitis media completed the validated Nepali Tinnitus Handicap Inventory. Severity was categorised using established grading systems. Associations between sex and severity were assessed using chi-squared analysis.

**Results:**

Nepali Tinnitus Handicap Inventory scores ranged from 0 to 94. Overall, 62 per cent of patients reported moderate or worse tinnitus, and 36 per cent reported severe or catastrophic symptoms. Female patients demonstrated a greater burden, with a higher proportion in severe and catastrophic categories. This difference was statistically significant (χ^2^ = 11.05; df = 4; *p* = 0.026).

**Conclusion:**

Patients undergoing surgery for chronic suppurative otitis media experience a substantial and under-recognised tinnitus burden. Routine assessment may support more comprehensive, patient-centred care.

## Introduction

Tinnitus is the perception of sound in the absence of an external stimulus and is commonly associated with hearing loss and chronic ear disease.^1^ Although often regarded as a secondary symptom, tinnitus may have a significant impact on quality of life, affecting emotional wellbeing, sleep, and daily functioning.^2^

Chronic suppurative otitis media (CSOM) remains highly prevalent in low- and middle-income countries, including Nepal. A population-based survey conducted by the Britain Nepal Otology Service reported hearing impairment in 16.6 per cent of the population and middle ear pathology in 7.4 per cent.^3^ Subsequent studies have confirmed a substantial burden of ear disease, particularly in children and young adults.^4^^–^^10^ However, these studies have largely focused on infection and hearing loss, with limited attention to associated symptoms such as tinnitus.

The Tinnitus Handicap Inventory (THI) is a validated instrument designed to assess the functional, emotional and catastrophic impact of tinnitus.^2^ A Nepali-language version (THI-N) has been developed and validated, demonstrating good reliability and internal consistency,^11^ and enabling structured assessment of tinnitus burden in this population.

The aim of this study was to evaluate the severity and impact of tinnitus in patients with CSOM presenting for surgical intervention in Nepal, and to explore differences in symptom burden within this cohort.

## Materials and methods

The THI-N was administered to patients attending the 66th Britain Nepal Otology Service (BRINOS) ear surgery camp at the BRINOS Ear Care Centre, Nepalgunj, Nepal, between 7 and 15 November 2022.

All patients were scheduled for tympanoplasty or mastoid surgery for chronic otitis media, with or without cholesteatoma, and had associated conductive or mixed hearing loss. Patients aged under 12 years were excluded. The questionnaire was completed on the morning of surgery. Where required, a trained Community Ear Assistant assisted illiterate participants by reading the questions aloud and recording responses.

THI-N scores were calculated according to standard methodology, generating a total score out of 100. Tinnitus severity was categorised as very mild, mild, moderate, severe, or catastrophic according to the grading system described by McCombe *et al*.^12^

Descriptive statistics were used to summarise demographic and clinical characteristics. Categorical variables are presented as frequencies and percentages. The association between sex and tinnitus severity category was assessed using the chi-squared (χ^2^) test. A *p*-value of less than 0.05 was considered statistically significant.

Tinnitus severity grading categories are defined in Table 1.

**Table 1.** Tinnitus grading according to McCombe *et al*. 2001^4^

Grading	THI score	Description
Very mild	0−16	Tinnitus perceived only in silence and easily masked; does not interfere with sleep or with daily activities.
Mild	18−36	Tinnitus is easily masked by environmental sounds and forgotten during daily activities; can occasionally interfere with sleep.
Moderate	38−56	Tinnitus perceived in the presence of environmental sound but daily activities not impaired. It is noticed less during concentration. Interferes with sleep and relaxation not infrequently.
Severe	58−76	Tinnitus is constant and hardly masked by external sound. It alters the sleep cycle and can interfere with daily activities. Relaxation is compromised.
Catastrophic	78−90	All side effects of tinnitus are present at a very severe level. Medical attention including neuropsychiatric help is frequently required.

THI-N = Nepali Tinnitus Handicap Inventory.

## Results

A total of 108 patients attended for surgery, of whom 100 completed the THI-N questionnaire. Eight patients were excluded due to age under 12 years. The study population had a mean age of 23 years (range, 13–58 years) and a female predominance (69 per cent female, 31 per cent male).

**Figure 1.**
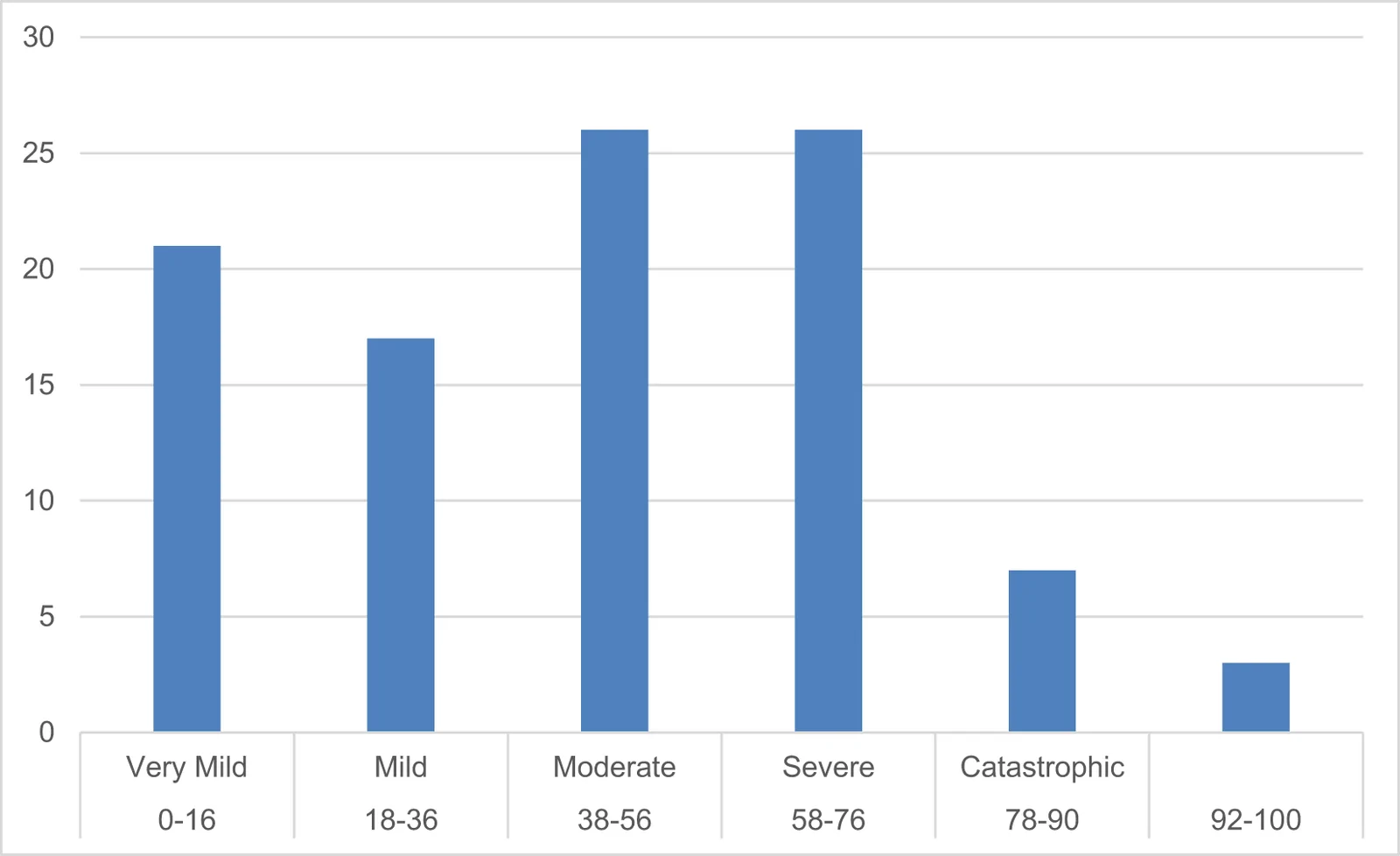
Number of patients in each category of tinnitus severity.

THI-N scores ranged from 0 to 94, demonstrating a wide distribution of tinnitus severity. The distribution of patients across severity categories is shown in Figure 1. Overall, 62 per cent of patients reported moderate or worse tinnitus, including 26 per cent with severe and 10 per cent with catastrophic symptoms.

**Figure 2.**
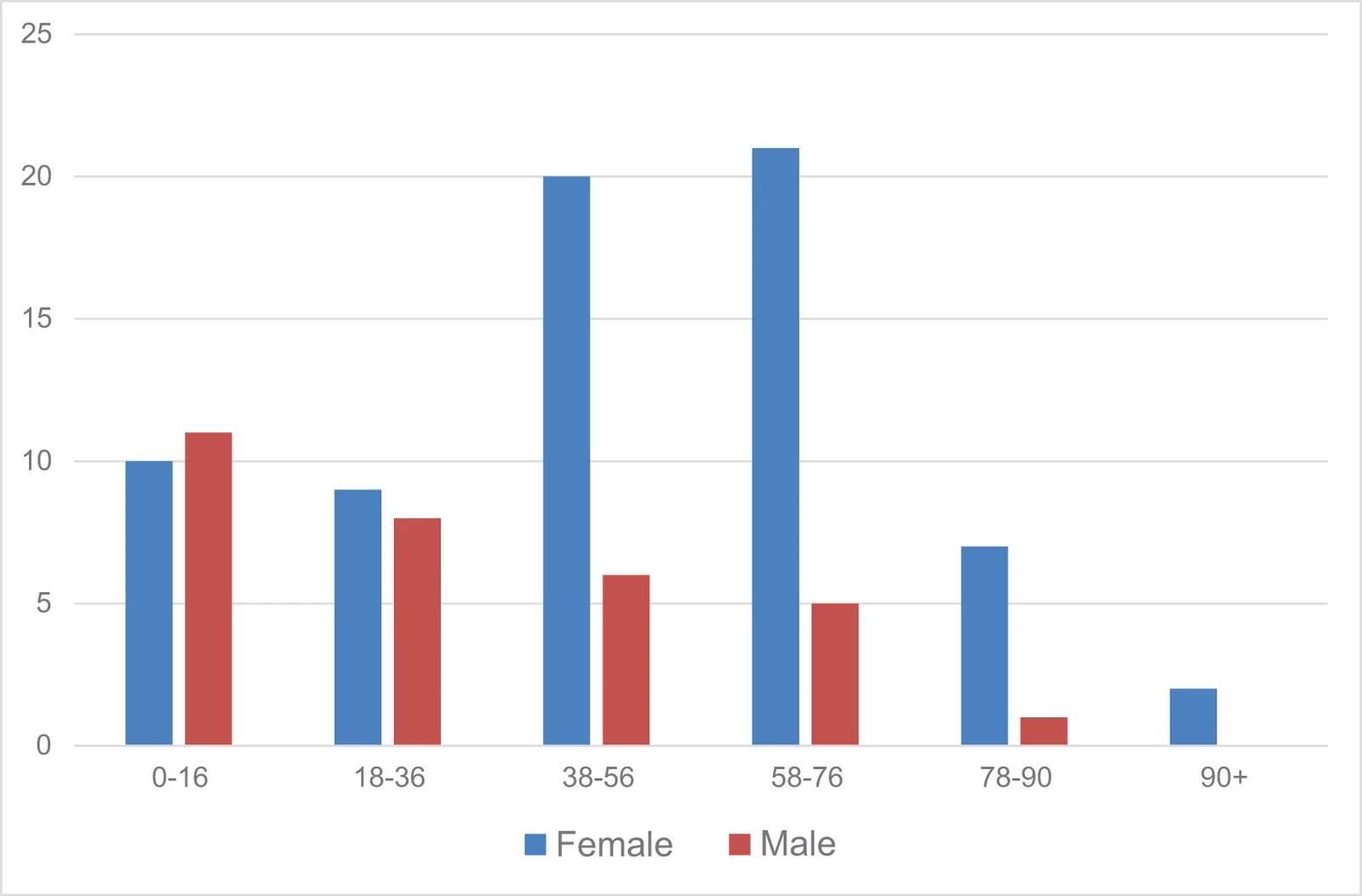
Nepali Tinnitus Handicap Inventory (THI-N) scores in male and female patients.

The distribution of tinnitus severity by sex is shown in Figure 2. Female patients demonstrated a greater burden of tinnitus, with a higher proportion falling into the severe and catastrophic categories compared to male patients. This difference in severity distribution was statistically significant (χ^2^ = 11.05; degrees of freedom = 4; *p* = 0.026).

**Figure 3.**
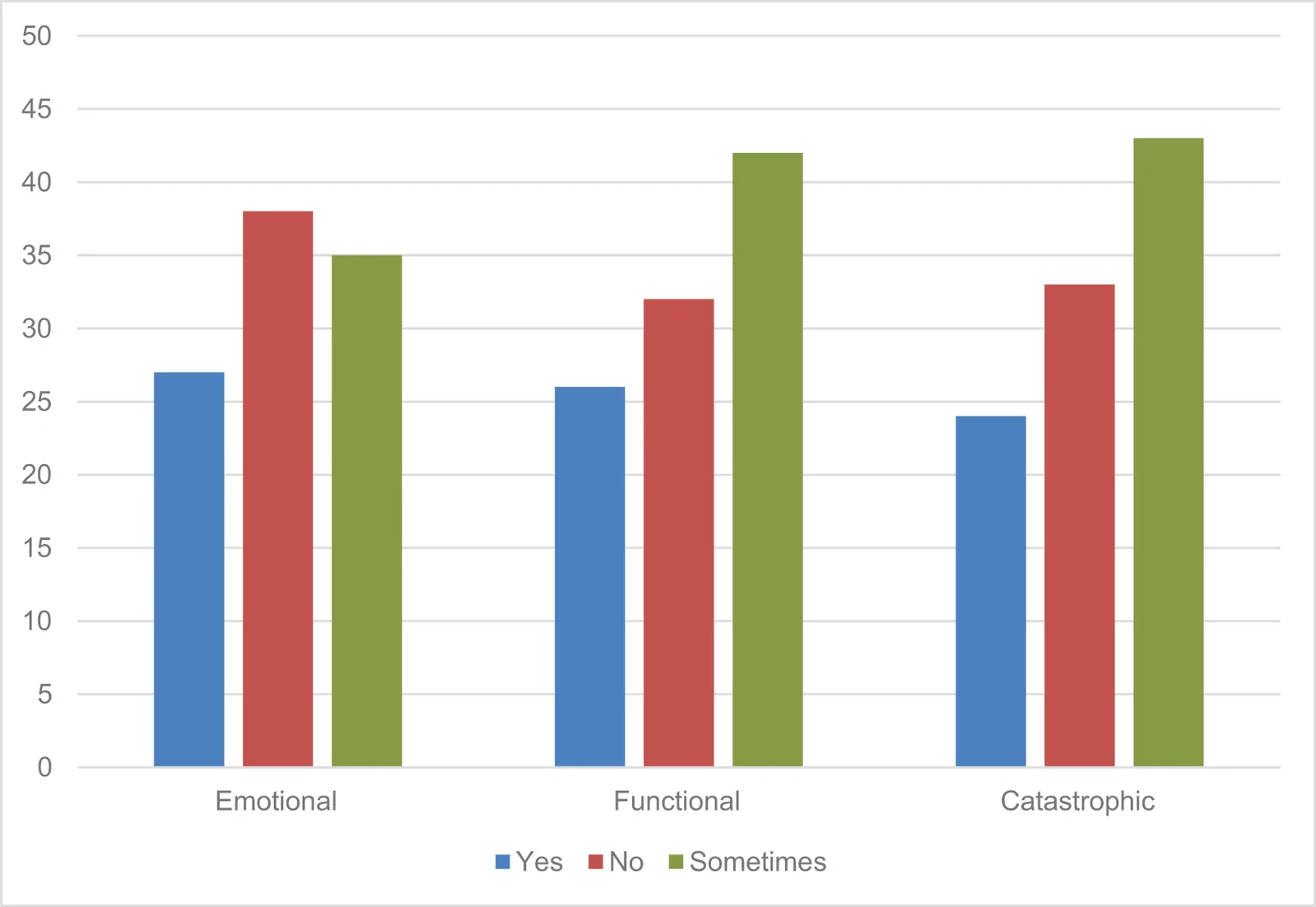
Percentage of individuals falling under the different subscales of the Nepali Tinnitus Handicap Inventory (THI-N).

The proportion of patients reporting symptoms within each THI-N subscale (emotional, functional and catastrophic) is shown in Figure 3, with 27 per cent, 26 per cent and 24 per cent of patients, respectively, reporting affirmative responses.

## Discussion

This study demonstrates a substantial burden of tinnitus among patients undergoing surgery for chronic suppurative otitis media in Nepal, with over one-third reporting severe or catastrophic symptoms (Figure 1). This highlights tinnitus as a clinically significant component of disease impact within this population, rather than a secondary or incidental symptom.

The distribution of severity observed in this cohort appears greater than that reported in previous studies of more general or mixed populations.^11^ This likely reflects the selected nature of the study group, comprising patients with established middle ear disease requiring surgical intervention. However, it also suggests that symptom burden may be under-recognised in clinical pathways that prioritise infection control and hearing restoration. Previous work in Nepal has demonstrated high levels of awareness of ear disease and hearing impairment but has not explored the impact of tinnitus in this context.^13^

A notable finding was the greater burden of tinnitus reported by female patients, with a significantly higher proportion falling into severe and catastrophic categories (Figure 2). This difference was statistically significant and suggests that sex-related factors may influence the experience or reporting of tinnitus. While the reasons for this are not fully understood, previous studies in other settings have suggested differences in symptom perception, coping strategies and psychosocial impact between male and female patients. This finding warrants further investigation but may have implications for patient counselling and support.

Subscale analysis demonstrated that tinnitus affected emotional, functional and catastrophic domains to a similar degree (Figure 3), indicating a broad impact on patients’ daily lives. This reinforces the importance of considering tinnitus as a multidimensional symptom, rather than focusing solely on its presence or absence.

The timing of questionnaire administration on the day of surgery may have influenced responses, as patients may have heightened awareness of their symptoms at this point in the care pathway. However, this may also reflect the real-world burden experienced at a critical stage in treatment and, therefore, remains clinically relevant.

This study has several limitations. The cohort represents a selected surgical population and is not intended to reflect population prevalence. In addition, the imbalance in sex distribution within the cohort may influence comparative findings. Data were analysed in aggregated form, limiting the scope of statistical analysis. Despite this, the use of a validated instrument and a clearly defined clinical population provides a useful insight into symptom burden in this setting.

Taken together, these findings suggest that tinnitus should be considered an important component of disease burden in patients with chronic suppurative otitis media. Incorporating simple, validated assessment tools such as the THI into surgical pathways may support more comprehensive and patient-centred care, particularly in lower-resource settings where such symptoms may otherwise be overlooked.

- The burden of tinnitus in patients with chronic suppurative otitis media in low-resource settings has not been well characterised
- In this surgical cohort in Nepal, 62 per cent of patients reported moderate or worse tinnitus, with over one-third reporting severe or catastrophic symptoms
- Female patients demonstrated a greater burden of tinnitus, with a significantly different severity distribution compared to male patients
- Tinnitus affected emotional, functional and catastrophic domains, indicating a broad impact on quality of life

## Conclusion

Patients undergoing surgery for chronic suppurative otitis media in Nepal experience a substantial burden of tinnitus, with a significant proportion reporting severe symptoms. This highlights tinnitus as an important component of disease impact that may not be routinely assessed in surgical pathways. Incorporating structured assessment of tinnitus may support more comprehensive and patient-centred care in this setting.

## Data Availability

Raw data are available from the first author on request.
